# Obesogenic dietary behaviours and work environment among primary healthcare workers in Limpopo Province: A cross-sectional analysis

**DOI:** 10.1177/22799036251388583

**Published:** 2026-07-31

**Authors:** Mashita Audrey Lehlohonolo, Mphasha Mabitsela Hezekiel, Skaal Linda

**Affiliations:** 1 Department of Public Health, University of Limpopo, Mankweng, South Africa; 2 Department of Public Health, Sefako Makgatho University, Garankuwa, South Africa

**Keywords:** obesity, workplace nutrition, dietary behaviour, food environment, obesogenic factors

## Abstract

**Background:**

Overweight and obesity are escalating public health challenges in South Africa. Healthcare workers (HCWs), expected to model healthy behaviours, are increasingly exposed to obesogenic environments, placing them at higher risk for poor dietary habits and obesity. This study investigated the prevalence of obesity and assessed dietary behaviours and workplace environmental influences among primary healthcare workers in Limpopo Province.

**Methods:**

A quantitative cross-sectional design was used. A total of 174 healthcare workers from clinics in Lepelle-Nkumpi Municipality participated. Stratified random sampling ensured representation across nursing, allied health, and support staff. Data were collected using a validated, self-administered questionnaire and Food Frequency Questionnaire. Anthropometric measurements were taken to calculate Body Mass Index (BMI). Data were analysed using SPSS version 28, with descriptive statistics and chi-square tests (*p* < 0.05) employed to explore associations.

**Results:**

Among participants, 76% were overweight (28%) or obese (48%), with significantly higher obesity prevalence among females (*p* = 0.001). A substantial proportion reported weekly fast-food consumption (50%), access to fast-food vendors near clinics (39.1%), and regular exposure to food advertisements (40.8%). Diets were characterized by frequent intake of full cream milk (64.9%), chicken with skin (53.4%), fried foods (47.7%), and low consumption of fruits and vegetables. Only 21.8% consumed traditional vegetables like spinach/morogo daily.

**Conclusion:**

This study reveals a high prevalence of overweight and obesity among healthcare workers, linked to unhealthy dietary patterns shaped by workplace food environments, fast-food access, and time constraints. Findings highlight the need for multi-level, culturally sensitive interventions and further exploration of home-based dietary influences.

## Background

Overweight and obesity are major global public health challenges, contributing significantly to rising rates of non-communicable diseases (NCDs) such as type 2 diabetes, cardiovascular conditions, and certain cancers. The World Health Organization (WHO) identifies obesity as a multifactorial condition influenced by behavioural, environmental, and socio-economic determinants.^1^ In recent years, low- and middle-income countries (LMICs) have experienced a sharp increase in obesity, driven by urbanization, reduced physical activity, and greater consumption of energy-dense, processed foods—an ongoing shift known as the nutrition transition.^2^

South Africa mirrors global obesity trends, with 68% of women and 31% of men classified as overweight or obese—rates highest among Black African women.^3^ This is largely linked to increased consumption of fast foods, sugary drinks, and processed snacks, coupled with reduced physical activity and a shift from traditional diets.^4^

Healthcare workers (HCWs) face a paradox: while expected to promote healthy lifestyles, they are exposed to the same obesogenic factors as the general population. Long hours, shift work, stress, and limited access to nutritious food contribute to poor dietary habits and elevated obesity risk.^5–8^ These challenges are especially acute in rural areas, where infrastructure and wellness support are often lacking.

The workplace significantly influences dietary behaviour, with factors like nearby fast-food outlets, limited healthy food options, and exposure to unhealthy food marketing contributing to poor eating habits.^9,10^ In healthcare settings, this creates a contradiction, as institutions promoting public health may neglect the health needs of their own staff, compromising both staff well-being and the credibility of health promotion messages.

In Limpopo Province, a predominantly rural and disadvantaged region, challenges to healthy living are intensified by poverty, food insecurity, and limited healthcare infrastructure, which hinder access to nutritious food and physical activity.^11^ Cultural beliefs linking larger body sizes to health and status, especially among Black African women, further complicate weight management efforts.^12,13^

Obesity in South Africa also reflects notable gender disparities. Women, who comprise the majority of the healthcare workforce especially in nursing are disproportionately affected by overweight and obesity. This is influenced by a combination of biological, behavioural, and social factors, including caregiving responsibilities, limited time for exercise, and societal expectations.^14^ This reality underscores the need for gender-sensitive approaches to workplace wellness.

Environmental and institutional factors create an “obesogenic environment” in healthcare settings, where lack of workplace policies supporting healthy eating and the availability of unhealthy food options promote poor dietary choices among HCWs.^15^ Without measures like affordable healthy food, scheduled breaks, and nutrition education, staff tend to choose convenient but unhealthy foods.

Addressing this issue calls for a shift from individual-focussed efforts to system-level strategies. Comprehensive workplace wellness programmes that create healthy food environments, supportive policies, and engage staff have been shown to improve behaviours and lower obesity risk.^16,17^ However, these interventions are still uncommon, especially in rural healthcare settings.

Although national obesity data are well documented, few studies have explored overweight and obesity among rural primary healthcare workers in Limpopo.^18,19^ Research often neglects the relationship between workplace environment, dietary habits, and obesity in this group. This study fills that gap by investigating obesity prevalence, related dietary practices, and workplace factors among rural HCWs to guide targeted, sustainable wellness interventions.

## Methodology

### Study design and approach

This study employed a quantitative, cross-sectional descriptive design to assess the prevalence of overweight and obesity, as well as to explore dietary behaviours and workplace environmental factors contributing to obesogenic risks among primary healthcare workers (PHCWs) in Limpopo Province, South Africa. This design allowed for the systematic collection and analysis of data at a single point in time, providing a snapshot of dietary patterns, body mass index (BMI), and work-related environmental influences without inferring causality.

### Study setting and population

The study was conducted in eight fixed clinics located in the Zebediela, Mphahlele, Mathabatha, and Mafefe areas within Lepelle-Nkumpi Local Municipality, part of the Capricorn District of Limpopo Province, South Africa. This municipality is predominantly rural, and these clinics serve as the first point of contact for healthcare services before referrals are made to three regional hospitals for specialized care. The clinics were purposively selected based on patient volume, geographical distribution, and accessibility, to ensure a representative sample across the municipality’s primary healthcare settings. The study targeted a multidisciplinary population of PHCWs employed in these clinics. Participants included professional nurses, enrolled nurses, allied health professionals (such as dietitians and physiotherapists), health promoters, administrative clerks, and support staff (e.g. cleaners and grounds personnel).

The inclusion criteria required that participants be permanently employed at the clinic for a minimum of 6 months, aged 18 years or older, workers of all genders, and directly involved in the day-to-day operations of the facility. Additionally, participants had to be available during the study period and willing to provide informed consent. Visiting or temporary staff were excluded from the study to ensure consistency in exposure to the clinic environment.

### Sampling procedure and sample size

Stratified random sampling was employed to ensure proportional representation across professional categories. The total population of healthcare workers in the municipality was 331. Using the Taro Yamane formula,^20^ a statistically appropriate sample size of 174 participants was calculated, allowing for a 5% margin of error: $S=\frac{n}{1+n{\left(0.05\right)}^{2}},$ where *S* = sample size, *n* = population size. Where *n* = sample size, *N* = population size (*N*=), *e* = error margin (5%), sample size = 331/1 + 331(0.05)^2^, *S* = 174.

### Data collection tools and procedures

Data collection was carried out between March and April 2023, after obtaining ethical clearance from the Turfloop Research Ethics Committee (TREC) and official permission from the Limpopo Department of Health. After information sessions, eligible participants gave written informed consent. Trained fieldworkers assisted in administering questionnaires and collected anthropometric data using standardized procedures to ensure data quality, confidentiality, and consistency.

The primary data collection instrument was a structured, self-administered questionnaire, developed through a comprehensive literature review and refined with input from registered dietitians and public health experts. A pilot study conducted at a non-participating clinic confirmed the tool’s reliability and no changes were necessary thereafter. The questionnaire consisted of the following four sections:

- Sociodemographic Profile

This section captured participants’ age, sex, marital status, job title, years of experience, and professional category (e.g. nursing, allied health, administration, or support staff).

- Anthropometric Measurements

Height and weight were measured using calibrated instruments. Body Mass Index (BMI) was calculated using the formula: BMI = weight (kg)/height (m²). BMI was then classified according to WHO standards^21^: Underweight: <18.5, Normal weight: 18.5–24.9, Overweight: 25.0–29.9, and Obese: ≥30.0.

- Obesogenic Workplace Environment

This section was adapted from the Obesogenic Environment Questionnaire,^22,23^ and assessed workplace factors influencing eating habits and weight status. Key areas included: Access to food (e.g. on-site tuckshops, vending machines, delivery services), Proximity to fast-food outlets, Availability of healthy food options, Workplace food marketing and advertisements, and Break time policies and designated eating areas. This tool aimed to assess the institutional and environmental contributors to obesogenic behaviours among HCWs.

- Dietary Intake Assessment

Dietary intake was assessed using a validated, semi-quantitative Food Frequency Questionnaire (FFQ) adapted for local relevance.^24,25^ The FFQ captured the frequency of food consumption over the past 30 days, using categories ranging from “never” to “daily.” This recall period was chosen to enhance accuracy and minimize recall bias. The FFQ included a wide range of food groups, with culturally and regionally relevant examples:

○ Protein sources: chicken (with/without skin), lean/fatty red meat, eggs, legumes, dairy,
○ Carbohydrate staples: maize meal, breakfast cereals, oats, porridge, rice, bread,
○ Fruits and vegetables: morogo, spinach, cabbage, carrots, apples, bananas,
○ Fats and spreads: butter, soft margarine, hard margarine, cooking oil,
○ Processed (industrially prepared foods with added salt, sugar, or preservatives) and fried foods: polony, samosas, vetkoek, fried chips, deep-fried meats,
○ Snacks and salty foods: crisps, biltong, popcorn, salted nuts.

Participants were also allowed to add any food items not listed in the FFQ that they consumed regularly.

### Data analysis

All collected data were carefully coded and entered into the Statistical Package for the Social Sciences (SPSS) version 28 for analysis. The dataset was first cleaned and checked for missing values, inconsistencies, and outliers to ensure accuracy and integrity. Descriptive statistics were employed to summarize the demographic characteristics of the participants, anthropometric measurements (including BMI categories), and dietary behaviours. Frequencies and percentages were used to describe categorical variables such as gender, professional category, dietary intake frequency, and exposure to obesogenic environments.

To explore potential relationships between BMI and key sociodemographic variables (such as gender, age group, marital status, and professional category), Pearson’s Chi-squared (χ²) tests were conducted. This inferential statistical method allowed for the evaluation of whether observed differences in BMI classifications across different groups were statistically significant. A *p*-value of less than 0.05 was considered statistically significant, and all tests were conducted at a 95% confidence level.

### Limitations and bias management

The study acknowledges potential recall bias, given the self-reported nature of dietary assessments. To mitigate this, participants were encouraged to report based on their recent dietary habits. Additionally, social desirability bias may have influenced responses; this was addressed through assurances of anonymity and non-judgemental data handling.

## Results

Table 1 shows a total of 174 African primary healthcare workers in Limpopo, most of whom were female (89%) and over 40 years old (66%). Nurses made up the majority (64%) of the workforce. A high prevalence of overweight and obesity was observed, with 76% of participants having a BMI above the normal range.

**Table 1.** Demographic and BMI Profile of Respondents (*n* = 174).

Variables	Categories	n	%
Gender	Female	154	89
	Male	20	11
Age (years)	≤40	60	34
	>40	114	66
Marital status	Unmarried	91	52
	Married	83	48
Race	African	174	100
Profession	Nursing	112	64
	Allied	8	5
	Support staff	57	31
BMI classification	Underweight	5	3
	Normal weight	37	21
	Overweight	49	28
	Obese	83	48

Table 2 presents the relationship between BMI classification and participants’ sociodemographic characteristics. A statistically significant association was found between BMI and gender (*p* = 0.001), indicating that females were more likely to be classified as obese compared to males. However, there were no statistically significant associations between BMI and age group (*p* = 0.168), marital status (*p* = 0.799), or professional category (*p* = 0.453).

**Table 2.** Cross-tabulation of BMI and sociodemographic characteristics of respondents (*N* = 174).

Socio demographic data		Body mass index classification				Chi-squared test and *p*-value
		Under weight	Normal weight	Overweight	Obese	
		N (%)	*N* (%)	*N* (%)	*N* (%)	
Gender	Males	0 (0)	11 (55)	7 (35)	2 (10)	*X*^2^ = 20.170, *p* = 0.001
	Females	5 (3.2)	26 (16.9)	41 (26.6)	82 (53.2)	
Age (years)	<40	4 (6.8)	15 (25.4)	17 (28.9)	23 (38.9)	*X*^2^ = 11.642, *p* = 0.168
	>40	1 (0.9)	22 (19.3)	30 (26.3)	61 (53.5)	
Marital status	Married	2 (2.4)	16 (19.0)	23 (27.4)	43 (51.2)	*X*^2^ = 1.657, *p* = 0.799
	Unmarried	3 (3.3)	21 (23.3)	25 (27.8)	41 (45.6)	
Professional category	Nurses	4 (3.6)	21 (18.7)	27 (24.1)	60 (53.6)	*X*^2^ = 7.799, *p* = 0.453
	Allied	0	2 (25)	2 (25)	4 (50)	
	Support staff	1 (1.8)	14 (25.9)	19 (35.2)	20 (37.1)	

Table 3 explores the food environment at participants’ workplaces. Approximately 39.1% reported the presence of fast-food outlets near their clinic entrances. A majority (60.3%) either had food delivered or had to walk to collect it. Additionally, 40.8% reported exposure to fast-food advertisements or visible menus at work. In terms of purchasing behaviour, only 5.2% reported daily purchases, while weekly and occasional purchases were more common at 50.0% and 44.8%, respectively.

**Table 3.** The frequency distribution of participants according to food availability, accessibility, advertisement, and marketing at work, *N* = 174, % in rows.

Work related obesogenic environment	Yes		No
Are there fast-food establishments located near the clinic entrance?	68 (39.1%)		106 (60.9%)
Is the food delivered to your location, or do you need to walk to pick it up?	69 (60.3%)		69 (39.7%)
Did the fast-food outlets advertise or show you their menus or products	71 (40.8%)		103 (59.2%)
On a monthly basis, how often do you go and buy their foods	Daily	Weekly	Occasionally
	9 (5.2%)	87 (50.0%)	78 (44.8%)

Table 4 shows that more than half (53.4%) consumed chicken with skin, while 43.7% opted for skinless chicken. Lean red meat (46%) was more popular than fatty cuts (42%). Full cream milk was the most consumed dairy option (64.9%).

**Table 4.** Consumption of the protein sources, *N* = 174, % rows.

Items	Category	Frequency	Percentages
Chicken	With skin	93	53.4
	Without skin	76	43.7
	None	5	2.9
Red meat	Fatty meat	73	42
	Lean meat	80	46
	None	21	12
Milk	2% or Low fat	34	19.5
	Skim/fat free	12	6.9
	Blends	9	5.3
	Full cream	113	64.9
	None	6	3.4

Table 5 indicates that soft margarine was the most commonly used spread (40.8%), followed by butter (29.9%). Almost half (47.7%) consumed fried foods weekly, and over half (54.6%) consumed processed foods occasionally or never.

**Table 5.** Use of spreads, fried and processed foods, *N* = 174, % rows.

Items	Category	Frequency	Percentages
Spread	Butter	52	29.9
	Hard margarine (brick)	16	9.2
	Soft margarine (Tub)	71	40.8
	None	35	20.1
Fried foods	Occasionally/never	80	46.0
	Weekly (at least once)	83	47.7
	Daily	11	6.3
Processed foods	Occasionally/never	95	54.6
	Weekly	66	37.9
	Daily	13	7.5

Table 6 reveals that most participants (81.6%) perceived their food as lightly salted. Over half (59.2%) never added extra salt, and 70.7% reported not eating salty snacks.

**Table 6.** Salt and salty snack consumption, *N* = 174, % rows.

Items	Category	Frequency	Percentages
Salted	Very salty	21	12.1
	Lightly salty	142	81.6
	Don’t know	11	6.3
Add salt/aromat	No, I never add salt	103	59.2
	Yes, but I taste first and then add	62	35.6
	Yes, even before having tasted food	8	4.6
	Do not know	1	0.6
Salty snack	Yes	51	29.3
	No	123	70.7

Table 7 shows that spinach/morogo was consumed daily by 21.8%, while 52.3% ate it 1–3 times per week. Apple consumption was also relatively common, with 26.4% consuming them daily. Several vegetables like green peas and butternut were consumed infrequently.

**Table 7.** Frequency of fruit and vegetable consumption, *N* = 174, % rows.

Consumption of fruit and vegetables	Never (%)	Weekly		Every day		
		1–3 times/week (%)	4–6 times/week (%)	1 time a day (%)	2 times a day (%)	3 times a day (%)
Spinach/morogo (traditional vegetable)	15 (8.6)	91 (52.3)	30 (17.3)	29 (16.7)	6 (3.4)	3 (1.7)
Tomato (raw/cooked)	39 (22.4)	76 (43.7)	25 (14.4)	26 (14.9)	4 (2.3)	4 (2.3)
Green peas	77 (44.3)	62 (35.6)	21 (12.1)	13 (7.5)	0 (0)	1 (0.6)
Mixed vegetables	50 (28.7)	74 (42.5)	29 (16.7)	13 (7.5)	3 (1.7)	4 (2.3)
Pumpkin/butternut	57 (32.8)	86 (49.4)	13 (7.5)	11 (6.3)	4 (2.3)	3 (1.7)
Sweet potato	72 (41.4)	74 (42.5)	12 (6.9)	9 (5.2)	5 (2.9)	2 (1.1)
Potato	35 (20.1)	90 (51.7)	30 (17.2)	15 (8.6)	4 (2.3)	0 (0)
Citrus fruit	27 (15.5)	75 (43.1)	37 (21.3)	25 (14.4)	3 (1.7)	6 (3.4)
Orange	71 (40.8)	66 (37.9)	21 (12.1)	11 (6.3)	4 (2.3)	1 (0.6)
Bananas	21 (12.1)	79 (45.4)	36 (20.7)	30 (17.2)	4 (2.3)	0 (0)
Mangoes	43 (24.7)	86 (49.4)	21 (12.1)	19 (10.9)	4 (2.3)	1 (0.6)
Apples	12 (6.9)	75 (43.1)	41 (23.6)	30 (17.2)	11 (6.3)	5 (2.9)
Avocado	76 (43.7)	66 (37.9)	17 (9.8)	12 (6.9)	2 (1.1)	1 (0.6)

Table 8 shows that over half (57.9%) of participants reported consuming breakfast cereal at least once per week. Soft porridge was consumed slightly less frequently, with 45.4% of participants indicating they never consumed it. Oat porridge had the highest rate of non-consumption, with half (50%) of the participants reporting they never consumed it for breakfast.

**Table 8.** Frequency of intake of carbohydrates for breakfast, *N* = 174, % rows.

Consumption of carbohydrate for breakfast	Never	Weekly		Everyday		
		1–3 times/week (%)	4–6 times/week (%)	Once (%)	2 times (%)	3 times (%)
Breakfast cereal	73 (41.9)	63 (36.2)	18 (10.3)	18 (10.3)	2 (1.1)	0
Oat porridge	87 (50.0)	57 (32.8)	18 (10.3)	9 (5.2)	3 (1.7)	0
Soft porridge	79 (45.4)	63 (36.2)	12 (6.9)	19 (10.9)	1 (0.6)	0

## Discussions

The purpose of this study was to determine the prevalence of overweight and obesity and to explore dietary habits and workplace environmental factors that may contribute to obesogenic risks among primary healthcare workers in Limpopo Province, South Africa. The demographic profile revealed that the majority of participants were female (89%).

The prevalence of overweight (28%) and obesity (48%) among HCWs in this study is alarmingly high, with approximately three out of four participants falling outside the recommended healthy weight range. These findings are particularly concerning given the occupational expectations for HCWs to serve as role models for healthy behaviours and to provide effective counselling on lifestyle modifications.^19,26^ Our findings closely align with a study conducted among healthcare workers in Mpumalanga, South Africa, where 51.9% of HCWs were classified as obese and 21.4% as overweight.^27^ This parallel suggests that obesity among HCWs may be a systemic issue within South Africa, especially in under-resourced or rural settings where environmental and occupational factors, including long shifts, work-related stress, and limited access to healthy foods at workplaces, may exacerbate weight gain.

However, our obesity prevalence appears to be higher than that reported in some international settings. For example, a study in Ghana reported an obesity prevalence of 28.9% and overweight prevalence of 22.7% among HCWs,^28^ while research in Nigeria found obesity rates of 27.3% and overweight prevalence of 44.7%.^29^ This comparison highlights that while obesity among HCWs is a shared concern across sub-Saharan Africa, the rates observed in our study and those in Mpumalanga are particularly elevated. Conversely, studies in high-income countries such as the United States report obesity rates among HCWs of 11.8% and overweight of 38.5%.^30^ This contrast underscores the potential influence of contextual factors such as socio-economic challenges, workplace environments, and broader public health infrastructure differences.

Notably, the current study found that female healthcare workers were significantly more likely to be obese than their male counterparts (*p* = 0.001). This gender disparity mirrors broader epidemiological patterns observed across sub-Saharan Africa, where socio-cultural norms, hormonal influences, and lifestyle behaviours contribute to higher obesity prevalence among women.^31^ Traditional perceptions of body image, especially among Black South African women, associate larger body sizes with beauty, wealth, fertility, and respectability.^32^ This cultural acceptance of larger body sizes may promote weight retention or gain, especially in rural contexts where such perceptions are deeply ingrained. Our findings are consistent with the Mpumalanga study, which also reported higher obesity rates among female HCWs,^19^ as well as studies in Nigeria,^29^ where female HCWs similarly exhibited higher obesity prevalence than males.

Furthermore, our findings reinforce the argument that interventions targeting obesity among HCWs should be both gender-sensitive and culturally appropriate. The marked gender disparity in obesity prevalence observed in this study highlights the need for strategies that consider the socio-cultural norms shaping body image and weight-related behaviours, particularly among women in rural South African contexts.^31,32^ In these settings, the unique interplay between work-related stress, entrenched cultural ideals, and food environments often characterized by limited access to affordable, healthy options calls for multi-level interventions that address both individual behaviours and the broader structural determinants of obesity.

From a public health perspective, these findings warrant urgent attention. Obesity among HCWs poses substantial individual health risks, including a heightened risk of cardiovascular diseases, type 2 diabetes, and musculoskeletal disorders.^33^ Beyond these personal health implications, excessive weight among HCWs may affect their work productivity, contribute to increased absenteeism, and potentially undermine the credibility of health promotion messages that they deliver to patients.^34,35^ Given their critical role as frontline advocates for healthy lifestyles, addressing obesity within this workforce is essential—not only for their well-being but also for ensuring the effectiveness of public health initiatives and the integrity of health education efforts.

The workplace food environment plays a pivotal role in shaping dietary choices and behaviours among employees. In this study, 39.1% of participants reported the availability of fast-food outlets near the clinic, while over 40% were regularly exposed to food advertisements or promotional menus. Although only 5.2% reported daily consumption of fast food, weekly consumption was notably high at 50%, indicating that unhealthy food options are routinely integrated into the eating habits of HCWs.

The obesogenic nature of the work environment identified in this study aligns with the concept of environments that promote weight gain through the easy availability, affordability, and marketing of energy-dense, nutrient-poor foods.^15^ The presence of fast-food outlets near healthcare facilities and the pervasive exposure to food marketing contribute to the normalization of unhealthy dietary patterns, even within settings charged with advancing public health.

Critically, this study exposes the contradiction between the health promotion mandate of healthcare institutions and the unhealthy food environments that often characterize these workplaces. Research has consistently shown that when HCWs are exposed to such environments, their own dietary behaviours may deteriorate, increasing their risk for overweight and obesity.^9,36^ This not only compromises the health of HCWs but may also undermine the credibility of their health promotion efforts directed at patients, especially when patients observe that HCWs themselves engage in the very behaviours they advise against. For instance, patients are more receptive to lifestyle advice from HCWs who visibly embody healthy practices.^37^

The frequent consumption of fast food among HCWs in this study raises critical questions about the structural and temporal demands of their work environments. Long shifts, high workloads, and limited protected breaks likely drive HCWs towards quick, convenient food options, often at the expense of nutritional quality.^9,38^ In contrast, workplace interventions in high-income countries, such as subsidized nutritious meals and protected meal breaks, have been shown to improve dietary choices among HCWs.^39^ These differences highlight the potential of policy-driven workplace reforms in reshaping food environments and supporting healthier choices in resource-limited settings.

Our findings suggest that the dietary patterns of HCWs lean heavily towards energy-dense, nutrient-poor foods, increasing their risk for overweight, obesity, and non-communicable diseases (NCDs). The frequent intake of high-fat meats, full-cream dairy, fried foods, and processed snacks is associated with excess saturated fat, sodium, and low dietary fibre—key contributors to poor cardiometabolic outcomes.^40,41^

In this study, 53.4% of participants reported consuming chicken with skin, and 42% consumed fatty red meats regularly, reflecting a high intake of saturated fats known to elevate low-density lipoprotein (LDL) cholesterol and increase cardiovascular risk.^42^ While animal-source foods provide vital nutrients like iron and vitamin B12, excessive consumption of fatty cuts and poultry skin can displace healthier protein options, such as legumes, fish, or lean meat. This eating patterns can be as a result of factors such as where cost, taste preferences, and convenience.^43^

The preference for full-cream milk (64.9%) over low-fat alternatives reflects not only taste preferences and cultural norms but also a potential lack of awareness regarding the benefits of lower-fat options.^44^ This is consistent with literature which noted low adoption of dietary guidelines among HCWs despite their expected role as health promoters.^45^

Similarly, while soft margarine (40.8%) was the most popular spread, its health advantage over butter depends on formulation. Some brands still contain trans fats or palm oil derivatives, which can adversely impact cardiovascular health.^46^

The high prevalence of weekly fried food (47.7%) and processed food (37.9%) consumption aligns with global evidence linking these dietary patterns to obesity, metabolic syndrome, and increased mortality.^47,48^ Conversely, interventions in Australian hospitals, including healthier cafeteria menus and vending machine regulations, significantly reduced fried and processed food intake among HCWs.^49^

Although the majority (81.6%) reported that their food was only lightly salted, a significant minority (35.6%) added salt after tasting, and 4.6% added salt before tasting. These behaviours are comparable to research which reported high discretionary salt use compounded already high sodium intakes from processed foods, contributing to hypertension prevalence.^50^ This adds to concern given that excessive sodium intake is a well-established risk factor for hypertension and cardiovascular disease,^51^ both of which are rising public health issues in South Africa.

Additionally, nearly 30% reported frequent consumption of salty snacks such as crisps, biltong, or popcorn. This is similar to patterns seen in peri-urban communities, where these snacks are often consumed as meal replacements rather than occasional treats, compounding the intake of sodium and unhealthy fats.^52^.

Perhaps most concerning is the low consumption of fruits and vegetables. While some traditional vegetables like spinach and morogo were consumed daily by 21.8%, most fruits and vegetables were consumed only weekly or less. This falls well short of the WHO-recommended daily intake of 400 g,^53^ and mirrors national trends showing insufficient fruit and vegetable intake among South African adults.^54^ In contrast, workplace initiatives healthcare settings that provided subsidized fruits and vegetables significantly boosted intake.^55^ The insufficient intake of these foods among HCWs contributes to micronutrient deficiencies (vitamin A, folate, fibre) that are critical for immune function and the prevention of NCDs.^56,57^

### Recommendations

#### Strengthen the workplace food environment

Introduce policies to limit unhealthy food vendors and advertising around healthcare facilities, regulate vending machines, and promote affordable, healthy food options within canteens.

#### Subsidize healthy meals

Provide subsidized, nutritious options such as fruits, vegetables, and wholegrain foods, particularly for staff on long or irregular shifts.

#### Implement protected meal breaks

Ensure HCWs have time and facilities to consume balanced meals, reducing reliance on fast foods.

#### Offer nutrition education

Deliver structured, practical education on healthy eating, meal preparation, and label reading, aligned with national dietary guidelines.

#### Explore home-work dietary links

Future research should assess how home-based dietary behaviours and the broader obesogenic environment influence workplace eating patterns and obesity risk among HCWs.

### Implications

Transforming healthcare workplaces into health-promoting environments can enhance HCW well-being, improve productivity, and strengthen the credibility of health promotion messages delivered to patients. Multi-level strategies that combine environmental, educational, and policy changes are vital. Institutional commitment to healthy procurement, staff involvement in food policy decisions, and fostering a culture that models healthy behaviours are essential to creating sustainable change.

## Conclusion

This study highlights a deeply concerning pattern of overweight and obesity among healthcare workers, with approximately three-quarters of participants falling outside the healthy weight range. These findings are particularly troubling given the critical role HCWs play as models of healthy behaviour and as providers of lifestyle counselling.

Our results further demonstrate that the workplace and surrounding food environment contribute meaningfully to unhealthy dietary patterns among HCWs. The proximity of fast-food outlets, frequent exposure to food advertisements, and the routine consumption of energy-dense, nutrient-poor foods—including high-fat meats, full-cream dairy, fried and processed foods—compound the risk of obesity and related non-communicable diseases.

The low intake of fruits and vegetables observed among participants points to a missed opportunity for nutrient-rich, protective diets in this population. Together, these findings reinforce the urgent need for multi-level interventions that improve the food environment, address structural barriers such as time constraints and workload, and promote sustainable healthy dietary practices among HCWs. Strengthening workplace policies, providing affordable healthy food options, and implementing culturally sensitive health promotion strategies are essential steps towards safeguarding both HCW well-being and the integrity of their role in advancing public health.

## Limitations

The cross-sectional design of this study limits the ability to draw causal inferences between workplace factors, dietary behaviours, and obesity outcomes. Associations observed may reflect correlation rather than causation, and longitudinal studies would be needed to establish temporal relationships. Dietary intake data were self-reported, which introduces the possibility of recall bias or social desirability bias. Participants may have underreported the consumption of unhealthy foods or overreported healthier choices, potentially skewing estimates of dietary patterns.

It is important to note that the study had a higher proportion of female participants compared to males, reflecting the gender composition of the healthcare sector, which is predominantly female. While this aligns with the workforce demographics, it may limit the ability to fully explore gender-based comparisons in dietary behaviours and obesity risk. Nonetheless, understanding these patterns in a female-saturated sector remains crucial, given the growing burden of obesity and the role of healthcare workers in modelling and promoting healthy lifestyles across all genders. The study did not present dietary intake patterns stratified by obesity classification or risk level (e.g. normal weight, overweight, obese). Such an analysis could have provided more nuanced insights into how dietary behaviours differ across weight categories and may have helped identify specific targets for intervention.

Other key factors that could significantly influence body weight, such as home-based obesogenic environment, physical activity levels, psychosocial stress, sleep patterns, and shift work, were not included in the current analysis. Future studies should aim to incorporate these variables to provide a more comprehensive understanding of the determinants of obesity among healthcare workers.
